# A Metagenomic-Based Approach for the Characterization of Bacterial Diversity Associated with Spontaneous Malolactic Fermentations in Wine

**DOI:** 10.3390/ijms20163980

**Published:** 2019-08-15

**Authors:** Carmen Berbegal, Luigimaria Borruso, Mariagiovanna Fragasso, Maria Tufariello, Pasquale Russo, Lorenzo Brusetti, Giuseppe Spano, Vittorio Capozzi

**Affiliations:** 1 Department of Agriculture, Food and Environment Sciences, University of Foggia, Via Napoli 25, 71122 Foggia, Italy; 2 EnolabERI BioTecMed, Universitat de València, 46100 Valencia, Spain; 3 Faculty of Science and Technology, Free University of Bozen-Bolzano, 39100 Bozen-Bolzano, Italy; 4 Istituto di Scienze delle Produzioni Alimentari, Consiglio Nazionale delle Ricerche, Unità Operativa di Supporto di Lecce, 73100 Lecce, Italy

**Keywords:** wine, malolactic fermentation, lactic acid bacteria, 16S rRNA metataxonomy, *Saccharomyces cerevisiae*, *Oenococcus oeni*, *Lactobacillus plantarum*, *Metschnikowia pulcherrima*, *Torulaspora delbrueckii*, malolactic consortium

## Abstract

This study reports the first application of a next generation sequencing (NGS) analysis. The analysis was designed to monitor the effect of the management of microbial resources associated with alcoholic fermentation on spontaneous malolactic consortium. Together with the analysis of 16S rRNA genes from the metagenome, we monitored the principal parameters linked to MLF (e.g., malic and lactic acid concentration, pH). We encompass seven dissimilar concrete practices to manage microorganisms associated with alcoholic fermentation: Un-inoculated must (UM), *pied-de-cuve* (PdC), *Saccharomyces cerevisiae* (SC), *S. cerevisiae* and *Torulaspora delbrueckii* co-inoculated and sequentially inoculated, as well as *S. cerevisiae* and *Metschnikowia pulcherrima* co-inoculated and sequentially inoculated. Surprisingly, each experimental modes led to different taxonomic composition of the bacterial communities of the malolactic consortia, in terms of prokaryotic phyla and genera. Our findings indicated that, uncontrolled AF (UM, PdC) led to heterogeneous consortia associated with MLF (with a relevant presence of the genera *Acetobacter* and *Gluconobacter*), when compared with controlled AF (SC) (showing a clear dominance of the genus *Oenococcus*). Effectively, the SC trial malic acid was completely degraded in about two weeks after the end of AF, while, on the contrary, malic acid decarboxylation remained uncomplete after 7 weeks in the case of UM and PdC. In addition, for the first time, we demonstrated that both (i) the inoculation of different non-*Saccharomyces* (*T. delbrueckii* and *M. pulcherrima*) and, (ii) the inoculation time of the non-*Saccharomyces* with respect to *S. cerevisiae* resources (co-inoculated and sequentially inoculated) influence the composition of the connected MLF consortia, modulating MLF performance. Finally, we demonstrated the first findings of delayed and inhibited MLF when *M. pulcherrima,* and *T. delbrueckii* were inoculated, respectively. In addition, as a further control test, we also assessed the effect of the inoculation with *Oenococcus oeni* and *Lactobacillus plantarum* at the end of alcoholic fermentation, as MLF starter cultures. Our study suggests the potential interest in the application of NGS analysis, to monitor the effect of alcoholic fermentation on the spontaneous malolactic consortium, in relation to wine.

## 1. Introduction

Wine is a complex hydro-alcoholic chemical mixture obtained via microbial-based bio-conversions of grape juice. A heterogeneous microbiota including, yeasts, bacteria, and filamentous fungi evolve from grape crushing up to the bottle, throughout oenological fermentations and processes [1]. Among the different microorganism, yeast has a predominant role in the metabolism of grape juice, which leads to the production of ethanol, and of other metabolites susceptible to influencing wine acceptance and quality, during the ‘alcoholic fermentation’ (AF) phase [2,3]. After the efficient development of yeasts, grape juice is converted to wine. A secondary desired microbial-driven process, the ‘malolactic fermentation’ (MLF), can take place in the must-wine system and mainly consists of the decarboxylation of malic acid in lactic acid performed by lactic acid bacteria (LAB) [4]. Also in this case, the microbial development is connected with the release of other metabolites, which are able to affect wine quality [5]. The model microorganisms for alcoholic and malolactic fermentation are, *Saccharomyces cerevisiae,* and *Oenococcus oeni*, respectively. The strains belonging to these two species have been selected to design starter cultures, which are useful for promoting AF and MLF in the wine sector [6,7,8]. However, a spontaneous consortium of microorganism is also associated with the matrix (grapes, must, and wine), and has been recently explored and characterized, in order to investigate their positive and negative roles, relating to specific oenological production [9,10,11,12]. With this concern in mind, in the last years, an increased interest has been raised in selecting species/strains belonging to the so-called non-*Saccharomyces* yeast species [13,14,15,16]. This attentiveness is attributable to the potential application of these resources to improve quality and/or safety of wine, and to the renovated interest in the study of microbial diversity connected with productions of specific *terroir* and susceptible to the influence peculiar organoleptic traits of regional wines [17,18,19]. A certain number of non-*Saccharomyces* strains have been already characterized and used to formulate commercial microbial preparations [8]. These starter cultures are often used in association with classical starter cultures to induce AF (i.e., *S. cerevisiae*) thereby diversifying wine production. Non-*Saccharomyces* species/strains are generally less adapted than *S. cerevisiae* to perform alcoholic fermentation, thus the management of these resources in enology foresee the use of a) co-inoculation with an increased concentration of the non-*Saccharomyces* or, b) a sequential inoculation with a preliminary inoculation of non-*Saccharomyces* [18,20,21]. While the reciprocal interactions among *S. cerevisiae* and the different non-*Saccharomyces* species/strains are explored [1,22,23], as well as the relationship among *S. cerevisiae* and the different bacteria connected to MLF [1,24,25], little is known about the impact of the use of non-*Saccharomyces* on the bacterial diversity belonging to the malolactic consortium and on the efficacy of malolactic fermentation [26,27,28,29]. Newly-developed sequencing strategies, usually known as next generation sequencing (NGS) techniques, involve versatile applications in the field of food microbiology, providing interesting insights in the study of microbiota related to food fermentation [30]. Among the other interesting application, NGS technique allowed advances into the description of bacterial diversity connected with wine-related environments [29,31]. In the last eight years, several investigations delved into prokaryotic diversity associated with grapes, grape musts, and/or grape wines (Table 1).

The widest part of these dealt with bacterial diversity on grapevines (Table 1) [32,34,40,41,43,44]. Some studies followed the evolution of bacterial communities up to the end of alcoholic fermentation [35,36,37,42,45]. Few works investigated the prokaryotic microbiota, associated with malolactic fermentation of wine [33,38,39] using NGS analysis, the biotechnological phase of the major positive impact of bacteria on wine quality [4]. Only in one case, in botrytized wine, the effect of spontaneous versus inoculated (*S. cerevisiae* biomass) alcoholic fermentation on the malolactic consortium was evaluated [33].

In this short communication, for the first time, we propose NGS analysis as a tool to monitor the effect of the varying ways to manage microbial resources associated with alcoholic fermentation on the spontaneous malolactic consortium, an issue that has attracted significant interest to ensure the quality and safety of wine fermentation. For the assessment of the performance, and of the bacterial diversity associated with the spontaneous malolactic consortium, we monitored the principal parameters of MLF (e.g., malic acid concentration, pH); and performed a phylogenetic analysis, based on 16S rRNA genes from the metagenome. Together with the simplest inoculated trial (*S. cerevisiae* biomass), we tested two cases of exploitations of indigenous yeast consortia corresponding to uninoculated must and on the practice of ‘*pied-de-cuve*’ in order to understand the influence of the different uses of microbial resources on malolactic fermentation. Considering the increasing interest in the use of selected non-*Saccharomyces* strains, our focus was on the use of non-*Saccharomyces* (*Metschnikowia pulcherrima* and *Torulaspora delbrueckii*) coupled with *S. cerevisiae* yeasts, using two different inoculation times (co-inoculation and sequential inoculation within one day). Finally, as a sort of further control trial, we compared these trials with the effect of adding starter cultures for MLF (i.e., *Oenococcus oeni*, *Lactobacillus plantarum*) at the end of alcohol fermentation.

## 2. Results

This paper reports the original findings of metagenomic-based approaches for the characterization of bacterial diversity associated with spontaneous malolactic fermentation in wine, integrated with some information about the classical monitoring of malolactic fermentation. The monitoring of the spontaneous malolactic consortium has been evaluated in relation to different uses of microbial resources, associated with the development of alcoholic fermentation in wine. In saying that, we also tested the use of bacterial starter cultures, that have been tailored to promote malolactic fermentation.

### 2.1. Experimental Modes and Monitoring of Malolactic Fermentation

The proposed experimental plan investigated the different ways to manage microbial resources associated with the alcoholic fermentation (Table 2; PdC, UM, SC, SCMPco, SCTDco, SCMPsq, and SCTDsq). The basic un-inoculation of the grape must (that means to rely on the spontaneous presence of indigenous yeasts) was compared with the practice of *pied-de-cuve*. *Pied-de-cuve* consists of the inoculation with a small amount of must, that was already undergoing spontaneous alcoholic fermentation [46] in a sort of microbial pre-enrichment practice. Hence, the fermentative process relies on the spontaneous presence of indigenous ‘enriched’ yeast. In all of the other experimental trials, the biomass of a single isolate from a commercial starter culture of *S. cerevisiae* was inoculated. In two experimental modes, this strain was combined with the biomass of a single isolate from a commercial starter culture of *M. pulcherrima*, using a co-inoculation and a sequential inoculation approach. In two other similar experimental modes, the *S. cerevisiae* strain was combined with the biomass of a single isolate from a commercial starter culture of *T. delbrueckii*, using co-inoculation and sequential inoculation methods. Finally, after *S. cerevisiae* development, in two trials, the biomass of single isolates from commercial starter cultures of *O. oeni* and of *L. plantarum* were inoculated separately, at the end of alcoholic fermentation (Table 2; SCOO, SCLP).

**Figure 1:**
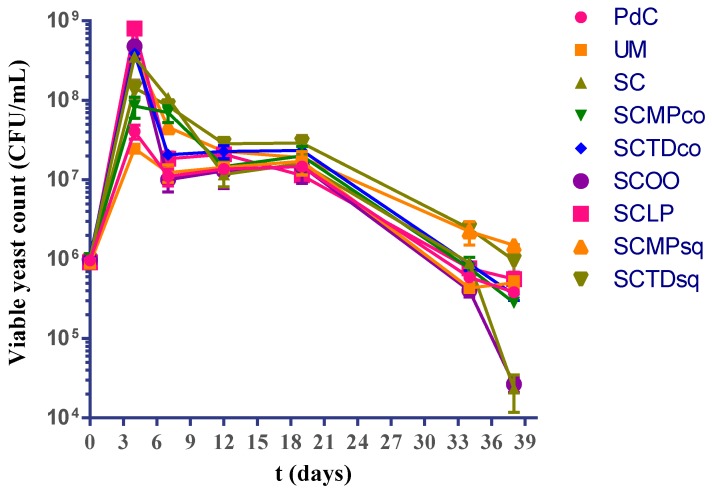
Variations of viable yeast during the vinification process in the different explored experimental modes: PdC: ‘*Pied-de-cuve*’; UM: Uninoculated must; SC: *S. cerevisiae*; SCMPco: *S. cerevisiae* + *M. pulcherrima* (co-inoculated); SCTDco: *S. cerevisiae* + *T. delbrueckii* (co-inoculated); SCOO: *S. cerevisiae* + *O. oeni*; SCLP: *S. cerevisiae* + *L. plantarum*; SCMPsq: *S. cerevisiae* + *M. pulcherrima* (sequentially inoculated); SCTDsq: *S. cerevisiae* + *T. delbrueckii* (sequentially inoculated). After 12 days of yeasts inoculation, biomass of *O. oeni* and *L. plantarum* has been added for the induction of malolactic fermentation in the trials SCOO and SCLP, respectively. The data shown are an average of three independent experiments.

In all the trials, total yeast counts were generally similar during alcoholic fermentation (Figure 1). The reduction of viable yeasts coincides with the third week after starting AF. Although, a more rapid decrease was noted in the samples where the malolactic fermentation started quickly.

**Figure 2:**
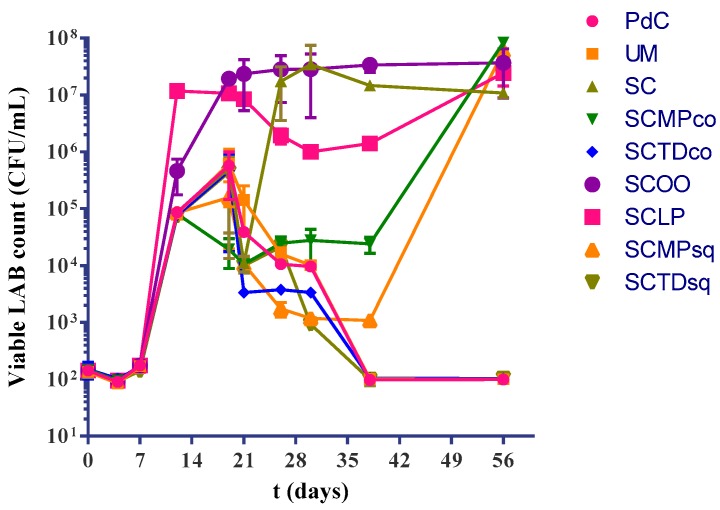
Variations of viable lactic acid bacteria during the vinification process in the different explored experimental modes: PdC: ‘*Pied-de-cuve*’; UM: Uninoculated must; SC: *S. cerevisiae*; SCMPco: *S. cerevisiae* + *M. pulcherrima* (co-inoculated); SCTDco: *S. cerevisiae* + *T. delbrueckii* (co-inoculated); SCOO: *S. cerevisiae* + *O. oeni*; SCLP: *S. cerevisiae* + *L. plantarum*; SCMPsq: *S. cerevisiae* + *M. pulcherrima* (sequentially inoculated); SCTDsq: *S. cerevisiae* + *T. delbrueckii* (sequentially inoculated). After 12 days of yeasts inoculation, biomass of *O. oeni* and *L. plantarum* was added for the induction of malolactic fermentation in the trials SCOO, and SCLP, respectively. The data shown are an average of three independent experiments.

The LAB count highlighted a considerable presence of malolactic bacteria associated with the samples, where a rapid malolactic fermentation was observed (SC, SCOO) (Figure 2). After inoculation of *L. plantarum* cells, viable bacteria was shown to slow down about ten-fold, and then raised to a concentration slightly higher than the inoculated one. The inoculation of *M. pulcherrima* led to a delay in the growth of LAB; in fact, only at the last sampling point (56th day), we found a concentration higher than 1 × 10^7^ CFU/mL, after a long latency at a concentration around 1 × 10^4^ CFU/mL and 1 × 10^3^ CFU/mL in the case of co-inoculation (SCMPco), and of sequential inoculation (SCMPsq), respectively. In the remaining fermentation trials (PdC, UM, SCTDco, SCTDsq), the LAB population decreased to less than 1 × 10^2^ CFU/mL after 38 days of incubation.

**Figure 3:**
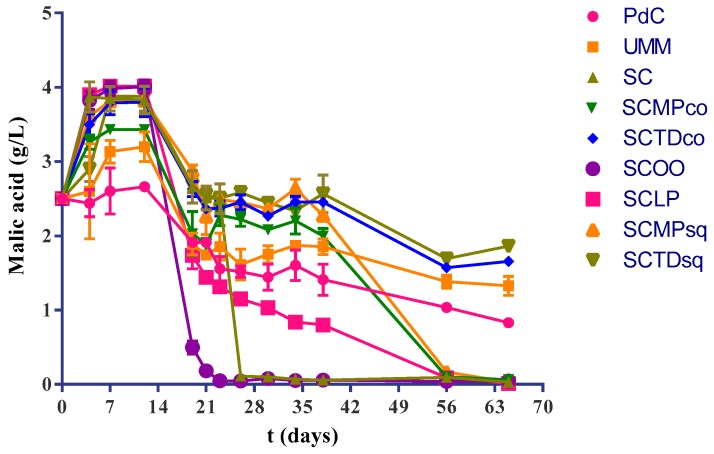
Malic acid concentration trends during the vinification process in the different explored experimental modes: PdC: ‘*Pied-de-cuve*’; UM: Uninoculated must; SC: *S. cerevisiae*; SCMPco: *S. cerevisiae* + *M. pulcherrima* (co-inoculated); SCTDco: *S. cerevisiae* + *T. delbrueckii* (co-inoculated); SCOO: *S. cerevisiae* + *O. oeni*; SCLP: *S. cerevisiae* + *L. plantarum*; SCMPsq: *S. cerevisiae* + *M. pulcherrima* (sequentially inoculated); SCTDsq: *S. cerevisiae* + *T. delbrueckii* (sequentially inoculated). After 12 days of yeasts inoculation, biomass of *O. oeni* and *L. plantarum* has been added for the induction of malolactic fermentation in the trials SCOO, and SCLP, respectively. In all the other experimental modes wines undergo spontaneous malolactic fermentation. The data shown are an average of three independent experiments.

Our findings highlighted complete malolactic fermentation after about three weeks (less than 2 weeks after the inoculation) when samples were inoculated with *O. oeni* after alcoholic fermentation (Figure 3). Moreover, the simple inoculation with *S. cerevisiae* alone led to a good performance of MLF (closed in little bit more than 2 weeks after the end of AF), while, the combination SCLP reduced the malic acid concentration to about 1 g/L in 2 weeks after inoculation. There was complete malic acid degradation in about 6 weeks after the end of the AF. In the samples inoculated with *M. pulcherrima* (i) MLF proceeded really slowly up to the fourth week after the end of AF, and (ii) a late but complete malolactic fermentation was observed (about 6 weeks after the AF). In contrast, an incomplete degradation of malic acid was observed in the remaining trials (UM, PdC, SCTDco, SCTDsq).

**Figure 4:**
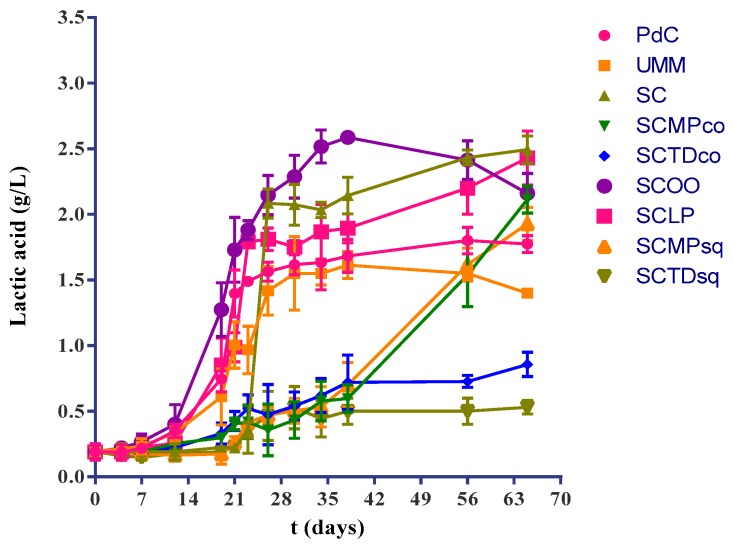
Lactic acid concentration trends during the vinification process in the different explored experimental modes: PdC: ‘*Pied-de-cuve*’; UM: Uninoculated must; SC: *S. cerevisiae*; SCMPco: *S. cerevisiae* + *M. pulcherrima* (co-inoculated); SCTDco: *S. cerevisiae* + *T. delbrueckii* (co-inoculated); SCOO: *S. cerevisiae* + *O. oeni*; SCLP: *S. cerevisiae* + *L. plantarum*; SCMPsq: *S. cerevisiae* + *M. pulcherrima* (sequentially inoculated); SCTDsq: *S. cerevisiae* + *T. delbrueckii* (sequentially inoculated). After 12 days of yeasts inoculation, biomass of *O. oeni* and *L. plantarum* has been added for the induction of malolactic fermentation in the trials SCOO, and SCLP, respectively. The monitoring started with the beginning of malolactic fermentation. The data shown are an average of three independent experiments.

Lactic acid productions was generally found symmetrical to the reported kinetics of malic acid degradation (Figure 4).

The comparison of the malic and lactic acid concentrations at the end-point (65 days, about 7 weeks after the end of AF) allowed us to highlight better the significant differences in the malolactic performances associated with the tested uses of the microbial resources (Table 3). The inoculation of *S. cerevisiae* is associated with an efficient consumption of malic acid. In contrast, the un-inoculated experimental modes (PdC, UM) led to consistent residual concentrations of L-malic acid (0.830 g/L, and 1.327 g/L respectively), even if we depicted a significantly better malic decarboxylation in the case of PdC management. With regard to non-*Saccharomyces* employment, the use of *M. pulcherrima* was found related with efficient malolactic fermentative performances, while inoculated *T. delbrueckii* cells resulted in unsatisfying levels of L-malic contents. Furthermore, in the case of *T. delbrueckii* addition, we can underline a slight, but significant difference between reached levels in L-malic content, in the case of co-inoculation and sequential inoculation with *S. cerevisiae* (1.657 g/L and 1.863 g/L).

**Figure 5:**
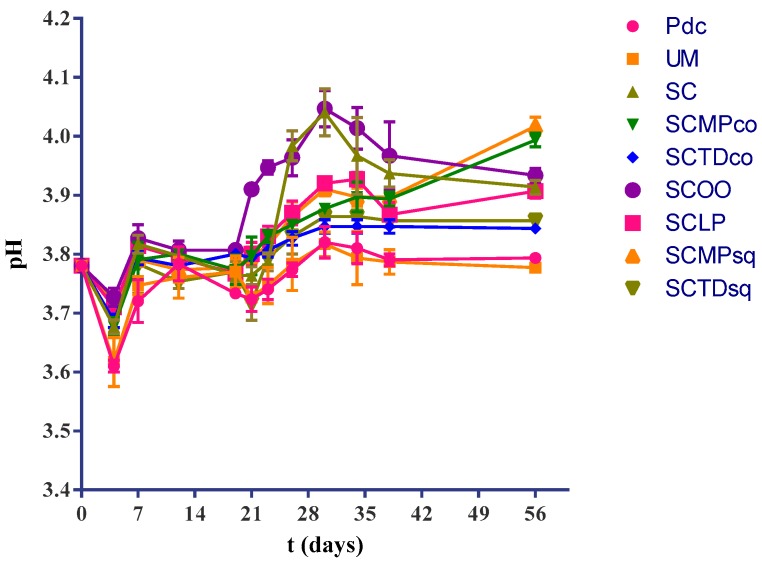
pH changes associated with the vinification process in the different explored experimental modes: PdC: ‘*Pied-de-cuve*’; UM: Uninoculated must; SC: *S. cerevisiae*; SCMPco: *S. cerevisiae* + *M. pulcherrima* (co-inoculated); SCTDco: *S. cerevisiae* + *T. delbrueckii* (co-inoculated); SCOO: *S. cerevisiae* + *O. oeni*; SCLP: *S. cerevisiae* + *L. plantarum*; SCMPsq: *S. cerevisiae* + *M. pulcherrima* (sequentially inoculated); SCTDsq: *S. cerevisiae* + *T. delbrueckii* (sequentially inoculated). After 12 days of yeasts inoculation, biomass of *O. oeni* and *L. plantarum* were added for the induction of malolactic fermentation in the trials SCOO, and SCLP, respectively. In all the other experimental modes, wine underwent spontaneous malolactic fermentation. The data shown are an average of three independent experiments.

We conducted the experiment using a must from the Apulian region (Southern Italy) characterized by a pH of about 3.8. This high pH is typical of hot climate zones of the Mediterranean area, and it is also representative of the possible chemical characteristics of grape must from other regions, related to global warming [47]. Considering the experimental findings, a slight deacidification, corresponding to a pH increase of about 0.2, was observed in the experimental modes SC, SCMPco, SCOO, SCLP, and SCMPsq (Figure 5). In contrast, only minor changes were found in trials PdC, UM, SCTDco, and SCTDsq. An increase of pH was firstly noted in the sample inoculated with *O. oeni*.

### 2.2. Malolactic Consortia Monitored using 16S High Throughput Sequencing

The analysis of 16S metagenomics was performed in all the experimental modes (Table 2) after 30 days of grape crushing, and the beginning of alcoholic fermentation during the MLF. Hence, the results provide ‘snapshot’ of the malolactic consortia after 30 days. This time had been chosen on the basis of, malic acid consumed, and lactic acid produced, as reported in Figure 2 and Figure 3, respectively. Moreover, the average time, reasonable for a spontaneous MLF in wineries, has been considered [48]. We waited a few days after that the first spontaneous MLF consortium completed malic acid degradation, remaining within the 3 weeks that are generally recognized as the average time for a spontaneous MLF in oenology. The bacterial diversity, associated with the malolactic consortia of the different experimental modes, was described by 16S rRNA gene metagenetic analysis. We obtained a total of 3,045,338 high-quality sequences of 16S rRNA, with an average of 88,376 ± 32,615 reads per sample. The total number of Operational Units (OTUs) was 17,540 with an average of 2573 ± 792 (Table S1).

**Figure 6:**
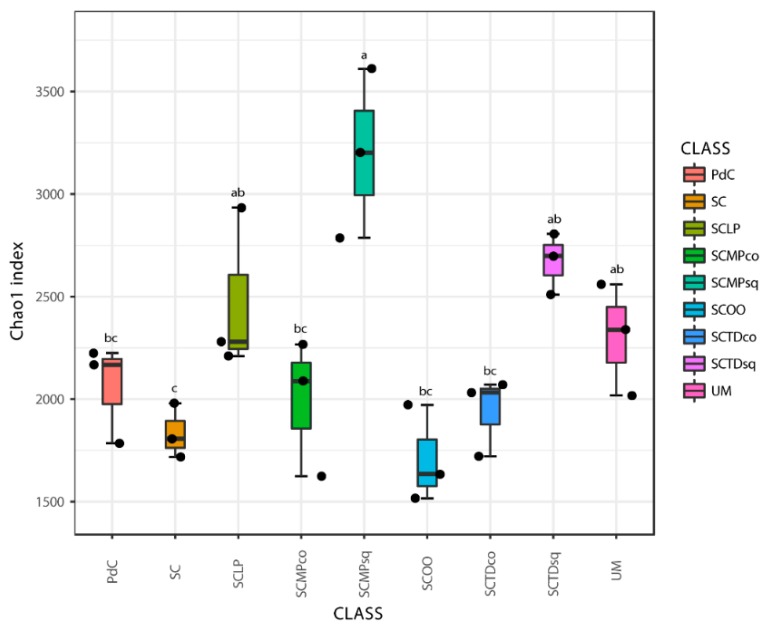
Alpha-Diversity estimates based on OTU-data with Chao1 indices for the experimental modes tested. Results of ANOVA analysis are shown as letters on the corresponding data.

**Figure 7:**
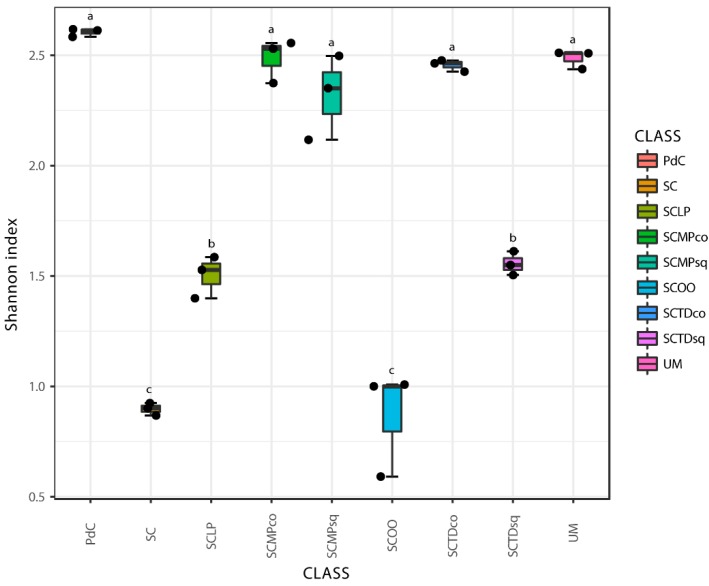
Alpha-diversity estimates based on OTU-data with Shannon indices for the experimental modes tested. Results of ANOVA analysis are shown as letters on the corresponding data.

Chao1 and Shannon indices (Figure 6 and Figure 7) were calculated for all the metagenomic malolactic consortia associated with the tested management of the microbial resources (Table 1). On the one hand, we report the calculation of the non-parametric richness estimator Chao1. On the other, we propose the values of Shannon’s diversity index that provide a measure of both richness and dominance.

Even if some trends appeared clear, poor significance in term of richness (Chao1 index), has been depicted (Figure 6). Only the combination of *S. cerevisiae* + *M. pulcherrima* sequentially inoculated to induce alcoholic fermentation led to a significantly richer malolactic bacterial consortium. On the opposite, richness and dominance (Shannon index) (Figure 7) were similarly highest for trials PdC, UM, SCMPco, SCTDco, SCMPsq, followed by the experimental modes, SCTDsq and SCLP. Finally, richness and diversity were significantly lower for samples inoculated with *S. cerevisiae* alone, and with *S. cerevisiae* and *O. oeni*.

**Figure 8:**
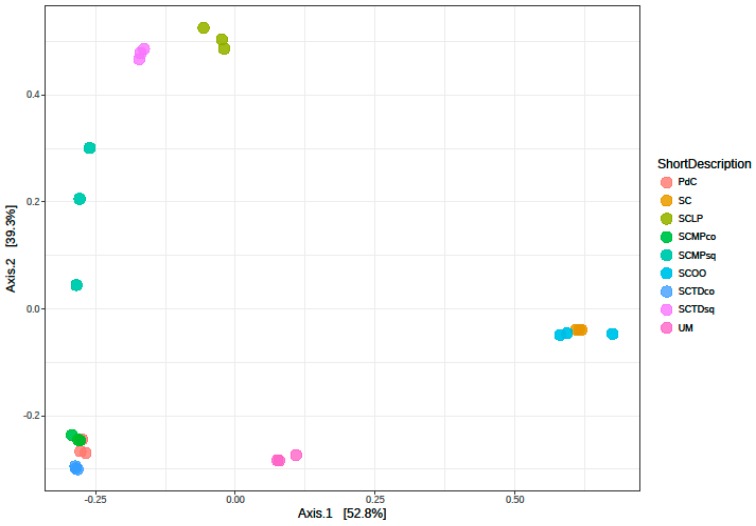
Principal Coordinates Analysis (PCoA) of the samples.

Another downstream output, generated from NGS data, is beta-diversity. Ordination plot in Figure 8, providing different cluster affinities, offers a measure of differences in bacterial community composition for the different malolactic consortia. PCoA demonstrated a clear separation of experimental mode clusters (Figure 8). The significance in the highlighted separation was proved by PERMANOVA tests among samples (*p* < 0.001). In particular, the three samples (PdC, SCTDco, and SCMPco) formed a separated thigh cluster. Two couples of samples, (i) inoculated with *S. cerevisiae* and with *S. cerevisiae* + *O. oeni,* and (ii) inoculated with *S. cerevisiae* + *T. delbrueckii* (sq) and *S. cerevisiae* + *L. plantarum*, respectively co-clustered. While uninoculated must, and samples inoculated with *S. cerevisiae* + *M. pulcherrima* (sq), formed separate congregations.

**Figure 9:**
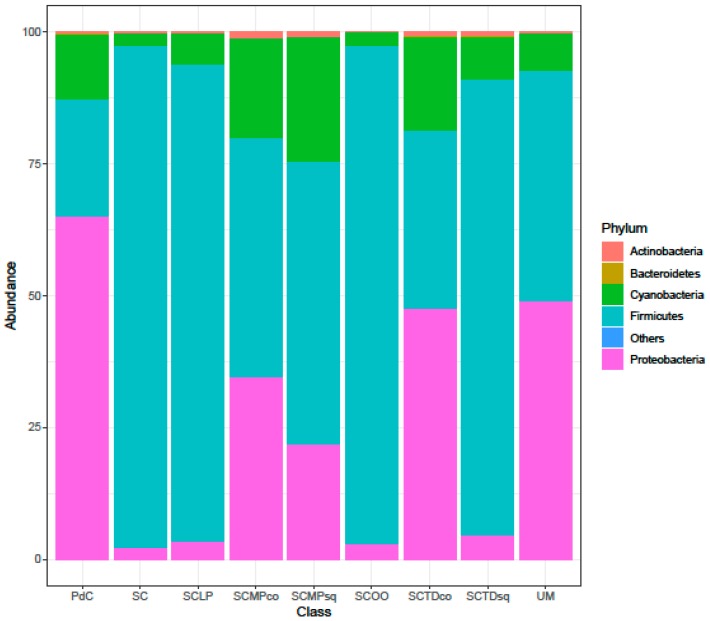
Bacterial community composition at the Phylum level of explored experimental modes. The data represent the means of three replicates. Results of ANOVA analysis are shown as letters on the corresponding data.

**Figure 10:**
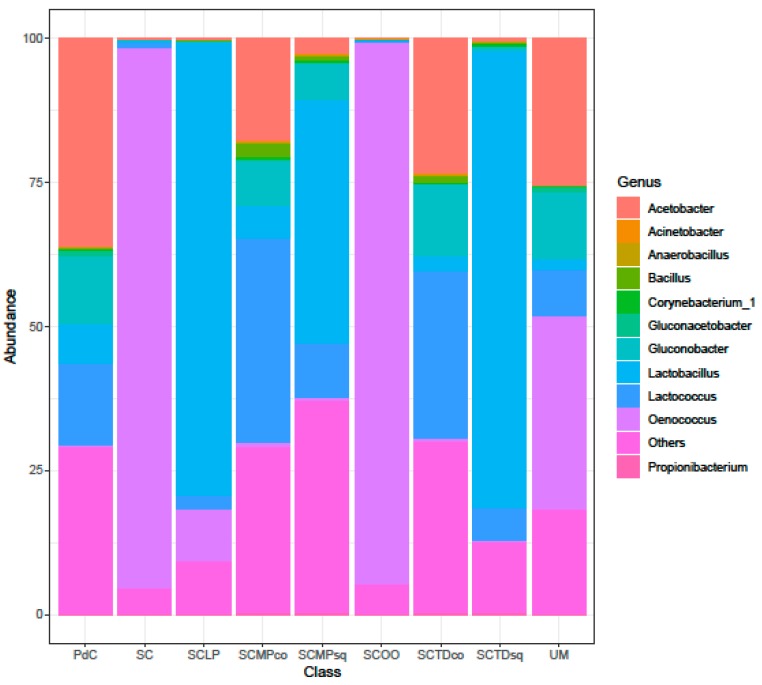
Bacterial community composition at Genus level of explored experimental modes. The data represent the means of three replicates. Results of ANOVA analysis are shown as letters on the corresponding data.

Considering the taxonomic composition of bacterial communities of the malolactic consortia, associated with the tested management of the microbial resources, Firmicutes were the dominant phylum in the experimental modes SC, SCTDsq, SCOO, and SCLP (Figure 9). This dominance was less pronounced in the samples SCMPsq and SCMPco. On the opposite, the increased relative abundance of Proteobacteria led to their dominance in the trials PdC, UM, and SCTDco (Figure 9). It is important to underline that, the presence of Cyanobacteria among the Phyla depicted would be ascribable to the amplification/sequencing of the 16S rRNA gene present in the DNA of *Vitis vinifera* chloroplasts [49] (a hypothesis strengthened by the evidence that it was not possible to assign with reasonable confidence Cyanobacteria OTUs at a finer than level Phylum). Diversity, at the genus level (Figure 10), was clearly in accordance with the diversity measured with α- and β-diversity (Figure 6, Figure 7 and Figure 8), providing information that can help explain the observed variability. It is possible to depict a certain correspondence between microbial consortium composition in terms of malolactic bacteria (genera *Oenoccoccus*, *Lactobacillus*, *Lactococcus*) (Figure 10) and the malolactic performances. This relation becomes clear in the case of dominance of *Oenococcus* genus.

**Figure 11:**
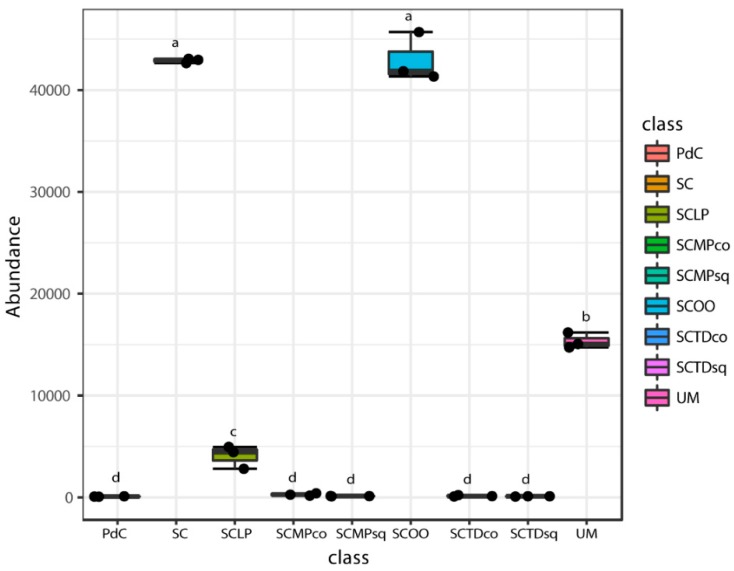
Boxplots showing the abundance of *Oenococcus* genus across tested experimental conditions. The data shown are an average of three independent experiments. Results of ANOVA analysis are shown as letters on the corresponding data.

**Figure 12:**
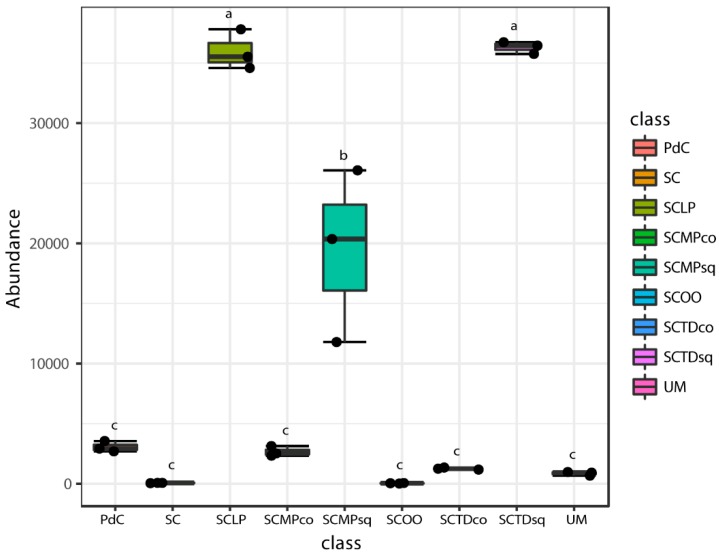
Boxplots showing the abundance of *Lactobacillus* genus across tested experimental conditions. The data shown are an average of three independent experiments. Results of ANOVA analysis are shown as letters on the corresponding data.

**Figure 13:**
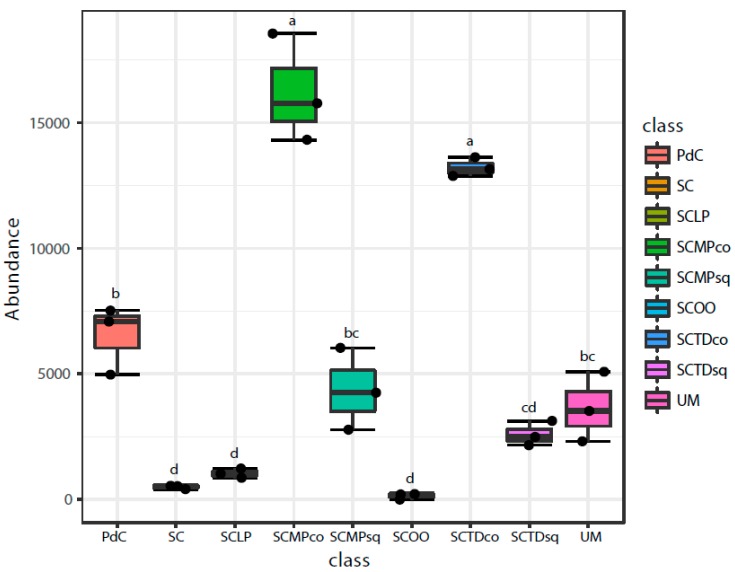
Boxplots showing the abundance of *Lactococcus* genus across tested experimental conditions. The data shown are an average of three independent experiments. Results of ANOVA analysis are shown as letters on the corresponding data.

**Figure 14:**
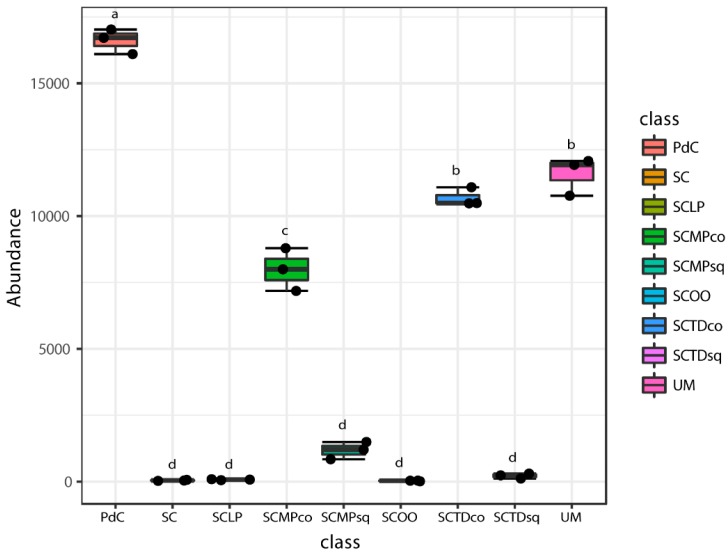
Boxplots showing the abundance of *Acetobacter* genus across tested experimental conditions. The data shown are an average of three independent experiments. Results of ANOVA analysis are shown as letters on the corresponding data.

**Figure 15:**
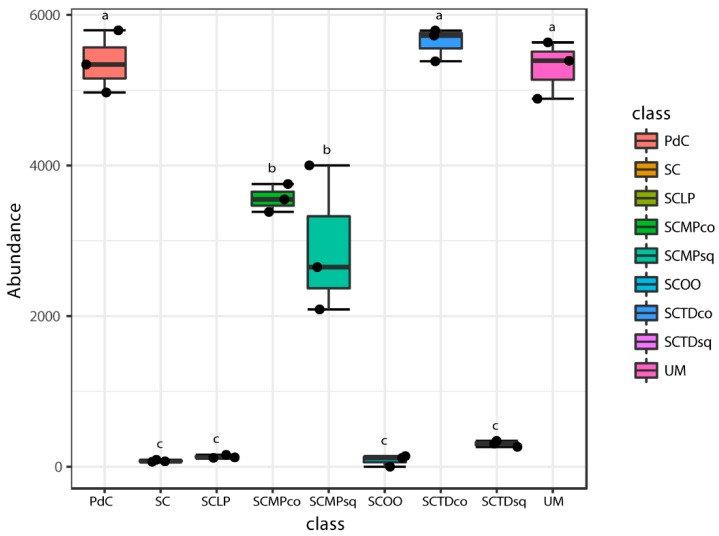
Boxplots showing the abundance of *Gluconobacter* genus across tested experimental conditions. The data shown are an average of three independent experiments. Results of ANOVA analysis are shown as letters on the corresponding data.

In Figure 11, Figure 12, Figure 13, Figure 14 and Figure 15, we report the 16S rRNA gene metagenomics data with the abundance of the number of reads for the principal bacterial genera with a pro-technological relevance [i.e., *Oenococcus* (Figure 11), *Lactobacillus* (Figure 12), *Lactococcus* (Figure 13)], and spoilage potential [i.e., *Acetobacter* (Figure 14), *Gluconobacter* (Figure 15)] in enology. In general, if we consider the more basic management of microbial resources related to alcoholic fermentation, ‘*pied-de-cuve*’ practice, un-inoculated must and inoculation of a selected strain of *S. cerevisiae*, we found that the genera of pro-technological significance were more abundant in the sample with a controlled alcoholic fermentation, followed by the uninoculated must, and by the ‘*pied-de-cuve*’ samples. In contrast, spoilage bacteria were more concentrated in the ‘*pied-de-cuve*’ sample, followed by the uninoculated trial. Comparing the experimental modes SC with the inoculation, in combination with a selected non-*Saccharomyces* strain, we found that inoculation with *T. delbrueckii*/*M. pulcherrima*, not only radically affects the composition of malolactic consortium, but we also found it to be associated with the prevalence of specific genera of oenological significance. In addition, it was also evident that the time of inoculation had an influence on the non-*Saccharomyces* commercial yeast. Specifically, within the pro-technological consortia, *Oenococcus* genus dominated the experimental mode SC and, to a lesser extent, by the ‘un-inoculated must’. *Lactobacillus* was detected as prevailing malolactic genus in the trial SCTDsq and SCMPsq, thus in the cases where the non-*Saccharomyces* strains were sequentially inoculated, with respect of the *S. cerevisiae* strain. In contrast, in the case of co-inoculation, the most representative LAB genus was *Lactococcus*, more so for sample SCMPco than in SCTDco. *Lactococcus* was also find to be significantly present in the sample where alcoholic fermentation was performed through the practice of ‘*pied-de-cuve*’. Considering spoilage bacteria of oenological significance, *Acetobacter* and *Gluconobacter* genera were found highly associated with uncontrolled alcoholic fermentations (UM and PdC), and to the experimental mode SCMPco, SCMPsq, and SCTDco (Figure 14 and Figure 15). Finally, we monitored the consortia associated with the inoculation of commercially available LAB, namely *Oenococcus oeni* and *Lactobacillus plantarum,* to promote malolactic fermentation. In both cases, we detected a considerable preponderance of the corresponding genus (*Oenococcus* in the sample SCOO and *Lactobacillus,* in sample SCLP), which suggests that the protechnological strains implanted well. Interestingly, in association with inoculation of the commercial *L. plantarum* strain, we were able to observe a significant presence of *Oenoccocus* genus.

## 3. Discussion

During the vinification process, we can generally recognize three main stages of interest, involving microorganisms: (1) Preliminary step of the AF associated with the dominance of the non-*Saccharomyces* yeasts, (2) the main phase of AF associated with the growth of *Saccharomyces* yeasts, and (3) MLF associated with the dominance of LAB. Wine quality strongly depends on the diversity of species and strains, linked to the microbial consortium, which continuously evolves during the process of winemaking [50]. The present work is one of the few studies dealing with the meta-taxonomic characterization of malolactic consortia in wine [33,38,39]. It is also the first study that proposes the application of NGS-based approaches, which explore the the different uses of eukaryotic resources, which have been exploited to drive the AF on the indigenous prokaryotic diversity, connected to malolactic fermentation. In addition, for the first time, we use a meta-taxonomic approach to monitor the effect of the inoculation of malolactic starter cultures after alcoholic fermentation. In particular, among the experimental modes, we tested different uses of microbial resources, commonly applied in wineries, relying (i) on spontaneous yeast consortium (un-inoculated must and ‘*pied-de-cuve*’ practices), and (ii) on the inoculation of selected pro-technological strains belonging to species, *Saccharomyces cerevisiae* [51,52], *Torulaspora delbrueckii* [20], *M. pulcherrima* [53], *O. oeni* [54], and *Lactobacillus plantarum* [55]. In all the explored experimental modes, malolactic fermentation, and the associated spontaneous consortium, were monitored in order to understand the phenomena (the management of microbial resources), which may be crucial to the quality of final wine [56]. In addition, the investigations of the autochthonous microbiota, linked to wine fermentation, and the relationship within the microbial consortium, are gaining increasing interest, even in light of the relevance of the so-called, ‘microbial *terroir*’ [19,34,38,57].

The classical analysis was performed to monitor malolactic fermentation, and it mainly looked at pH, malic acid degradation, lactic acid production, and microbial counts, which were, in general, coherent. Together with the direct proportion among malic acid degradation, lactic acid production, and lactic acid bacteria level, even the slight deacidification of about 0.2 in terms of pH, clearly indicate the occurrence of MLF [57]. Our data indicate no consistent malic acid degradation by the non-*Saccharomyces* strains used (*T. delbrueckii*, *M. pulcherrima*). This was in accordance with recent evidence that, studying different non-*Saccharomyces* strains found only *Schizosaccharomyces pombe* and the *Candida zemplinina* responsible for a mentionable degradation of this organic acid [28,58].

Concerning malolactic performances, a first relevant observation deals with the efficiency of spontaneous malolactic fermentation, connected to the inoculation of *S. cerevisiae,* when compared to un-inoculated and ‘*pied-de-cuve*’ samples. Our evidence confirms that, what was found in common wine corresponds with the finding related to botrytized wine by Bokulich et al. [33]: Comparing the bacterial diversity between uninoculated and inoculated trials, it is possible to notice a selective pressure exerted by *Saccharomyces*, mainly in the reduction of acetic acid bacteria. In addition, we found that this reduction in the genera, *Acetobacter* and *Gluconobacter* is associated with an opposite trend in the relative abundance of the genus *Oenococcus*. In this context, PdC sample showed behavior that appeared intermediate between UM and SC samples, in terms of *Acetobacter*, *Gluconobacter,* and *Oenococcus* relative abundance. The variations in malic consumption, followed the *Oenococcus* relative abundance, confirmed the relevance of this genus in the wine environment, and its significance in terms of MLF performances [59]. Taken together, our finding suggests that the control AF inoculating *S. cerevisiae* can favor the dominance of *Oenococcus*, reducing acetic bacteria, and improving MLF [24]. We also clearly indicated the limitations in terms of MLF efficiency in the case of uninoculated must and of ‘*pied-de-cuve*’, underlining the risks linked to vinification processes that rely on spontaneous AF [60,61].

Regarding the impact of non-*Saccharomyces* yeast on the spontaneous malolactic consortium, we report, for the first time, a potential inhibition/delaying of MLF when *T. delbrueckii* and *M. pulcherrima* were inoculated. Although our study does not suggest any further information on the possible biological mechanisms responsible for these findings (e.g., nitrogen source preferences, the release of medium chain fatty acids) [26,62], evidence from the recent literature, underline that a possible solution relies on the use of selected starter cultures for MLF. The use of selected resources to drive MLF in wine allowed the possible evaluation of the compatibility of the selected malolactic bacteria, with specific non-*Saccharomyces*, as recently reported for *O. oeni* strains, used in combination with *T. delbrueckii*, *M. pulcherrima* and *Hanseniaspora uvarum* strains [28,63,64,65]. Intriguingly, we also show an effect of the inoculation time of the non-*Saccharomyces* strains with respect to *S. cerevisiae* resources, which is a fundamental aspect in the concrete winery practices [66].

Furthermore, the preponderance of the genera *Oenococcus* and *Lactobacillus,* in association with inoculated and spontaneous MLF, was confirmed [67,68], as well as a potential role for the genus *Lactococcus* [69,70].

All reported findings highlight the importance in applying optimal yeast management and the MLF strategy to ensure correct completion of MLF and, consequently, to improve wine quality. Furthermore, these preliminary findings suggest that massive sequencing could be a new useful tool for the control malolactic fermentation, not only for the monitoring of AF in wine [71]. The findings highlight the importance of selecting the appropriate sampling point for the NGS analysis, in order to elucidate the composition of the microbial consortia, associated with malic acid degradation. In this light, the monitoring of more time points represents a crucial issue among the future perspectives of this study.

Considering fermented matrices as experimentally tractable microbial ecosystems [72], our study corroborate the potential interest in wine as a model matrix. In fact, there are two temporarily separated fermentative processes (AF and MLF), with the opportunity to study the effects of a perturbation of the first microbial ecosystem (this associated with the alcoholic fermentation) on the second one (this associated with the malolactic fermentation).

## 4. Materials and Methods

### 4.1. Microorganisms

The following microorganisms were used for must inoculation: The commercially available *S. cerevisiae* DV10 (Lallemand Inc., Montréal, QC, Canada) [73], the commercially available non-*Saccharomyces* strains *Metschnikowia pulcherrima*, and *Torulaspora delbrueckii*. As lactic acid bacteria (LAB), *Oenococcus oeni* and *Lactobacillus plantarum* were used in the fermentation. Starter cultures have been purchased in active dried form. Rehydration procedures were carried out according to suppliers’ instructions, and single isolates were selected on agarized YPD medium (2% glucose, 2% Bacto peptone, 1% yeast extract) and in MRS (Sharlab, Barcelona, Spain) at 28 °C (incubated for 2 days; 5 days for *O. oeni*) for yeasts, and bacteria, respectively. The biomasses used as starter cultures were prepared by growing up to the stationary state the yeasts strains separately in liquid YPD medium and LAB in MRS (Sharlab, Barcelona, Spain) at 28 °C.

### 4.2. Vinifications

Starter cultures were prepared by growing strains in YPD or MRS medium, as described above, and then inoculating the strains into 400 mL of grape must from the Apulian autochthonous grape varieties ‘Uva di Troia’ (21° Babo; 7.2 g/L total acidity; 2.57 g/L malic acid; pH 3.78). Each fermentation experiment was carried out by performing three simultaneous independent repetitions into sterile 1 l bottles, with an air lock and fermented at 20 °C. Using the 3 yeasts (in a different inoculation mode) and 2 LAB, a total of 9 different starter culture combinations were carried out (Table 2). In mixed yeast starter cultures, *S. cerevisiae* was inoculated simultaneously with the non-*Saccharomyces* yeast in a 1:100 proportion (10^4^ CFU/mL:10^6^ CFU/mL) or sequentially at the same cell concentration 24 h later. Lactic acid bacteria were inoculated (10^6^ CFU/mL *O. oeni*; 10^7^ CFU/mL *L. plantarum*) after 12 days of yeasts inoculation, when AF was finishing. One uninoculated grape must sample and an inoculated grape must sample, by a “pied de cuve”, were used as controls. The kinetics of the fermentation was monitored for 40 days. During this time, L-malic acid, lactic acid and pH variation were determined by enzymatic kits (Biogamma, Rome, Italy), and with a pH meter, respectively.

### 4.3. Determination of Microbial Population

The viable count of yeasts during the AF was enumerated on YPD agar medium (Sigma-Aldrich, Saint Louis, MO, USA). Plates were incubated at 28 °C for 48 h. The viable count of LAB was done in MRS (Sharlab) supplemented with 10 mg/L cycloheximide (Sigma-Aldrich, Saint Louis, MO, USA) and incubated for 4–7 days at 28 °C.

### 4.4. Genomic DNA Extraction

Samples of 5 mL from the wine fermentations, incubated during 30 days, were centrifuged (10000 rpm, 5 min), washed with ultrapure water (1 vol), then with 1 vol of 10% TEN buffer (0.1M Tris-HCl pH 7.5, 0.05 M EDTA, 0.8 M NaCl) supplemented with polyvidone 10 (PVP) (2% *w*/*v*) (Sigma-Aldrich, Saint Louis, MO, USA), and then twice with ultrapure water (1 vol). The genomic DNA from the 5 mL sample, after the washing steps, was extracted with the commercial extraction kit, known as PowerSoil Microbial DNA Isolation Kit (Mo Bio, Carlsbad, CA, USA), according to the manufacturer’s instructions.

### 4.5. High Throughput Sequencing

Illumina libraries were prepared using a NEXTflex 16S V4 Amplicon-Seq Kit (Bioo Scientific, Austin, TX, USA). Briefly, from 50 ng of DNA template for each sample, the bacterial V4 region of the 16S rRNA gene was amplified using the universal primers V3F (GTGCCAGCMGCCGCGGTAA) and V4R (GGACTACHVGGGTWTCTAAT) tailed with Illumina barcoded adapters under the following touchdown PCR conditions: A 20 μL mixture was prepared for each reaction and included a 1 × reaction buffer (TAKARA), 2 mM Mg^2+^, 0.2 mM dNTP, 0.1 μM of each primer, 1 U HotStarTaq polymerase (TAKARA) and 2 μL template DNA. The cycling programme was: 95 °C for 2 min; 35 cycles of 94 °C for 20 s, 55 °C for 40 s, 72 °C for 1 min, 72 °C for 2 min. PCR products were purified using Agencourt XP AmpureBeads (Beckman Coulter, Brea, CA, USA). The quality of the final products was assessed using a Agilent Bioanalyzer 2100 (Agilent Technologies, Palo Alto, CA, USA). After their quantification with Qubit (Invitrogen, Carlsband, CA, USA), the samples were pooled in equal proportions and sequenced paired-end in an Illumina MiSeq platform (San Diego, CA, USA) with 312 cycles (150 cycles for each paired read and 12 cycles for the barcode sequence) at IGA Technology Services (Udine, Italy). A tailored pipeline has been adopted in order to process raw sequencing data from 16S rRNA gene amplicon sequence analysis. We used the software PEAR [74] to merge paired-end reads from each library. We avoided including sequences with a quality score threshold, lower than 30 and shorter than 200 bp, and we processed the assembled reads with QIIME v.1.8 software [75], inspecting for chimeras with the Chimera VSEARCH package [76]. Subsequently, we taxonomically annotated the SILVA reference database [77]. Operational Units (OTUs) were generated with 97% similarity cutoff using demultiplexed sequences, removing singletons. The operational taxonomic units (OTUs) table was generated using at 97% similarity. To normalize the variation in-read depth across samples, data were rarefied to the minimum read depth of 45,747 sequences per sample.

### 4.6. Statistical Analysis and Estimation of Fungal Alpha and Beta Diversity

Statistical analysis has been performed using R software [78]. Alpha and Beta diversity analyses were performed using the phyloseq and vegan packages [79,80]. Differences in Chao1, Richness, and Shannon values among the samples were tested using ANOVA and Pairwise Tukey test. Principle Coordinate Analysis (PCoA), based on Bray–Curtis, was used to displaying beta-diversity. The statistical significance of the clustering pattern visualized via PCoA was tested using Permutational ANOVA (PERMANOVA). Differences among the most important Genera (>1%) was investigated via ANOVA and Pairwise Tukey test.

## Figures and Tables

**Table 1:** List of studies on bacterial diversity associated with grapes and/or musts and/or wines.

Year	Subject	References
2011	Study of bacterial communities associated with the leaf and berry surfaces of ‘Chardonnay’ grape	[32]
2012	Study of bacterial communities associated with botrytized wine fermentations (sampling time compatible with MLF)	[33]
2014	Grape-associated microbial biogeography from different regions of California	[34]
2015	Study of bacterial communities associated with spontaneous wine fermentations (from six Portuguese wine appellations) in the initial musts, start and end of alcoholic fermentation	[35]
2015	Influence of the use of sulfur dioxide (in winemaking) on bacterial diversity	[36]
2015	Study of the evolution of bacterial communities during the alcoholic fermentation of organically and conventionally produced wines	[37]
2016	Study of the bacterial communities associated with the main wine fermentation stages including malolactic fermentation	[38]
2016	Study of the bacterial communities in berries, musts, and wines, including malolactic fermentation	[39]
2016	Study of the bacterial communities associated with Corvina berries at the end of the withering process performed in two different conditions	[40]
2018	Correlation between soil- and grape-associated bacterial communities in vineyards	[41]
2018	Study of bacterial diversity associated with healthy, rotten, botrytized grapes and with the derived musts and wine (up to the alcoholic fermentation)	[42]
2018	Study of the epiphytic bacterial community of vine bark and its relationships with grape bacterial diversity	[43]
2019	Study of bacterial diversity associated with the grape surface of samples collected from different wine regions in Xinjiang, China	[44]
2019	Bacterial communities associated with alcoholic fermentation, including the variation between musts that successfully complete alcoholic fermentation and those that become ‘stuck’ in the process	[45]

**Table 2:** List of the experimental modes tested in our experimental plan. Each microbial management explored has been tested in three independent biological replicates.

Codes	Tested Management of The Microbial Resources
PdC	‘*Pied-de-cuve*’ practice
UM	Uninoculated must
SC	*S. cerevisiae*
SCMPco	*S. cerevisiae* + *M. pulcherrima* (co)
SCTDco	*S. cerevisiae* + *T. delbrueckii* (co)
SCOO	*S. cerevisiae* + *O. oeni*
SCLP	*S. cerevisiae* + *L. plantarum*
SCMPsq	*S. cerevisiae* + *M. pulcherrima* (sq)
SCTDsq	*S. cerevisiae* + *T. delbrueckii* (sq)

co: co-inoculated; sq: sequentially inoculated.

**Table 3:** L-malic acid and lactic acid concentration at the end of the vinification process (day 65) in the different explored experimental modes. Results of ANOVA analysis are shown as letters (a–f) on the corresponding data.

	L-malic Acid (g/L)	Lactic Acid (g/L)
PdC	0.830 ± 0.052 ^a^	1.773 ± 0.064 ^a^
UM	1.327 ± 0.127 ^b^	1.400 ± 0.001 ^b^
SC	0.030 ± 0.017 ^c^	2.493 ± 0.105 ^c^
SCMPco	0.062 ± 0.032 ^c^	2.113 ± 0.102 ^d^
SCTDco	1.657 ± 0.021 ^d^	0.857 ± 0.093 ^e^
SCOO	0.046 ± 0.020 ^c^	2.160 ± 0.151 ^d^
SCLP	0.013 ± 0.005 ^c^	2.430 ± 0.206 ^cd^
SCMPsq	0.300 ± 0.017 ^c^	1.927 ± 0.127 ^ad^
SCTDsq	1.863 ± 0.025 ^e^	0.530 ± 0.005 ^f^

## Supplementary Materials

Supplementary materials can be found at https://www.mdpi.com/1422-0067/20/16/3980/s1.

## Abbreviations

AF	Alcoholic Fermentation
MLF	Malolactic Fermentation
LAB	Lactic Acid Bacteria
OTUs	Operational Units
NGS	Next Generation Sequencing
