# Supplementary material for: A Metagenomic-Based Approach for the Characterization of Bacterial Diversity Associated with Spontaneous Malolactic Fermentations in Wine
Source: Int J Mol Sci. 2019 Aug 15;20(16):3980. doi: 10.3390/ijms20163980 (PMC6721008; doi:10.3390/ijms20163980)
Supplement: Supplementary file 1 [file ijms-20-03980-s001.pdf]

Table S1

| Sample | Quality filtered bacterial reads (OTU table based) | |
|---|---|---|
| #NAME | #reads | #OTU |
| v0a | 54146 | 1892 |
| v0b | 75162 | 2301 |
| v0c | 74177 | 2446 |
| v1a | 87215 | 2783 |
| v1b | 60639 | 2255 |
| v1c | 88091 | 2897 |
| v2a | 94515 | 2292 |
| v2b | 66736 | 1755 |
| v2c | 64960 | 1753 |
| v3a | 88919 | 2696 |
| v3b | 45888 | 1665 |
| v3c | 79135 | 2401 |
| v4a | 47746 | 1705 |
| v4b | 67181 | 2184 |
| v4c | 69253 | 2292 |
| v5a | 81562 | 2067 |
| v5b | 53544 | 1697 |
| P5c | 91139 | 1865 |
| v6a | 160761 | 3974 |
| v6b | 80618 | 2419 |
| v6c | 80385 | 2477 |
| v7a | 152296 | 4403 |
| v7b | 90992 | 2982 |
| v7c | 154230 | 4399 |
| v8a | 106244 | 3015 |
| v8b | 126865 | 3120 |
| v8c | 143755 | 3744 |

| **Counts/sample summary** | | | |
|---|---|---|---|
| Reads | | OTU | |
| Total count | 3045338 | Total count | 17540 |
| Min | 45888 | Min | 1665 |
| Max | 160761 | Max | 4403 |
| Median | 80618 | Median | 2401 |
| Mean | 88376 | Mean | 2573.3 |
| Std. dev. | 32615 | Std. dev. | 792 |
